# Atrioventricular Conduction Disorders as a Complication of Inferior ST-Elevation Myocardial Infarction in Patients with COVID-19 Infection

**DOI:** 10.1155/2022/3621799

**Published:** 2022-12-12

**Authors:** Yaser Alahmad, Hassan Al-Tamimi, Fadi Khazaal, Mawahib Elhassan, Abdulrahman Arabi, Jassim Al Suwaidi, Awad AlQahtani, Nidal Asaad

**Affiliations:** Hamad Medical Corporation, Heart Hospital, Doha, Qatar

## Abstract

This case series demonstrates how COVID-19 infection might affect the heart in the context of acute myocardial infarction. Atrioventricular (AV) block might appear as one of the significant cardiac complications of acute MI in patients who tested COVID-19 PCR positive regardless of the presence of CVOID-19 infection symptoms. In our series, conduction disorders as a complication of acute inferior STEMI are more common in patients who tested positive for the COVID-19 infection. 11 patients out of 18 inferior STEMI patients who have tested positive for the COVID-19 infection have atrioventricular block disorders.

## 1. Introduction

Coronavirus disease 2019 (COVID-19) is a viral infectious disease caused by severe acute respiratory syndrome coronavirus 2 (SARS-CoV-2). It first emerged in Wuhan, China, in December 2019 [1].

Multiple studies have shown that elders, especially those with chronic underlying diseases, including chronic pulmonary disease, heart diseases, and diabetes mellitus, are more susceptible to COVID-19 complications [2]. Various types of complications with different prevalence have been reported in literature consisting of neurologic [3], hematologic (including coagulative disorders) [4], hepatic [5], renal [6], and finally cardiovascular complications [7].

Multiple studies have shown both increased susceptibility in patients with underlying cardiovascular disease to COVID-19 and severe cardiovascular complications in COVID-19 infected patients, including acute myocardial injury, thrombotic events, arrhythmias, and shock with challenges in acute management [8, 9, 10].

The type and severity of atrioventricular (AV) block associated with COVID-19 have not been described. We aimed to evaluate the incidence of the AV block as a complication, angiographic findings, and clinical outcome in the setting of acute inferior STEMI in patients with COVID-19 infection. We noted that the incidence of atrioventricular block as one of the complications of acute inferior STEMI is higher in patients who were tested as COVID-19 positive versus COVID-19 negative cases.

COVID-19 infection was confirmed with reverse transcription-polymerase chain reaction assays (PCR). Inferior STEMI was defined based on the presence of typical cardiac symptoms associated with at least 1 mm ST-segment elevation in inferior leads.

A total of 44 patients were admitted with acute inferior STEMI between May 1, 2020 and June 15, 2020. All patients met the definition criteria of STEMI with localized ST-elevation, and all were treated in the setting of emergent activation. 18 patients (40.9%) had tested positive for the COVID-19 infection, while 26 patients (59.1%) had tested negative for the COVID-19 infection (59.1%). No complications related to atrioventricular conduction disorders were documented in non-COVID-19 infection patients who presented with acute inferior STEMI (0%). While 11 out of 18 positive COVID-19 infection patients had atrioventricular block (AV) disorders (61%), 8 patients had a high degree of atrioventricular block (44.5%), and 3 patients developed first-degree AV block (16.6%). Although all patients shared the same basic characteristics, including age, gender, race, demographic distribution, and risk factors, conduction disorders were significantly higher in inferior STEMI with positive COVID-19 infection tests.

In this special case series, we are going to present all cases of high-degree atrioventricular block (AV) and one of the three cases of first-degree AV block as the one with the longest PR interval.  Table 1 demonstrates the summary of the case series.

This data was collected from Qatar cardiovascular COVID-19 registries. It was approved by the Institutional Review Board in Hamad Medical Corporation, and all performed procedures were following institutional guidelines and recommendations. Due to the nature of the study, written informed consent was not required. The authors declare that all supporting data are available within the article.

## 2. Case 1

**Figure 1:**
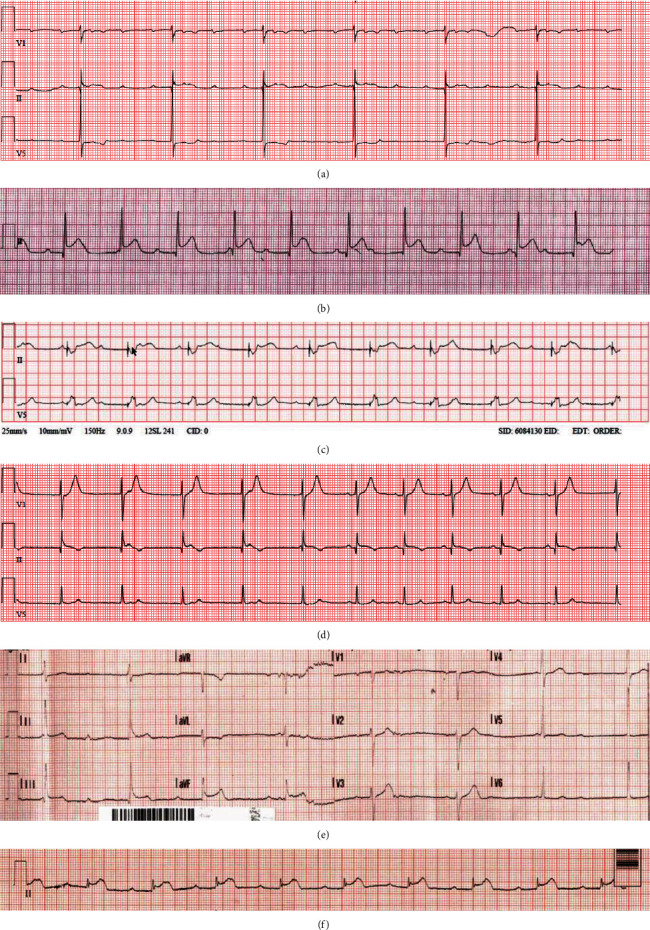
(a) Case 1: strip lead II and V5 showed significant sinus bradycardia 40 per minute with 3 : 1 high-grade AV block. (b) Case 2: strip lead II showed ST-segment elevation in lead II with complete heart block. (c) Case 3: strip lead II and V5 revealed pacing rhythm with complete heart block. (d) Case 4: strip leads V1, II, and V5 demonstrate junctional rhythm and isorhythmic AV dissociation. (e) Case 7: ECG showed ST-segment elevation in inferior leads with complete heart block. (f) Case 9: strip lead II shows ST segment with first-degree AV block PR = 340 milliseconds.

**Figure 2:**
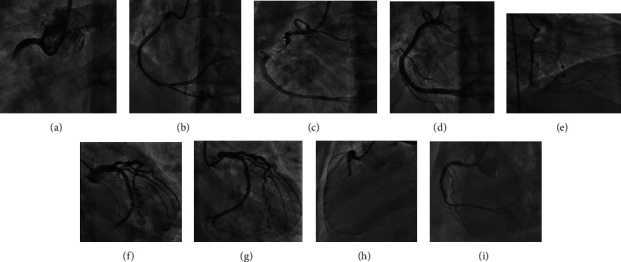
Coronary angiography to the culprit lesion. Case 1: (a) left anterior oblique (LAO) projection for right coronary artery (RCA), a thrombotic occlusion to proximal segment; (b) successful PCI to RCA with drug-eluting stent DES, and distal microembolization to very distal RCA happened with TIMI II flow distally. Case 2: (c) left anterior oblique (LAO) projection for right coronary artery (RCA) severe thrombotic stenosis in the proximal segment with TIMI II flow; (d) successful thrombus aspiration and PCI was done to RCA with one drug-eluting stent DES, and TIMI III flow was achieved. Case 3: (e) an attempt with balloon angioplasty was done to the thrombotic stenosis in mid-RCA but failed to achieve TIMI III flow. Case 4: (f) caudal projection showing 100% thrombotic stenosis in distal left coronary circumflex LCx; (g) successful PCI to distal LCx with one drug-eluting stent (DES) with TIMI III flow. Case 5: (h) total mid-RCA occlusion; (i) successful PCI to mid-RCA with one DES insertion.

A 48-year-old male complained of chest pain associated with dizziness, subsequently, he shortly lost consciousness and injured his face. He was taken by his colleagues to the peripheral hospital, upon arrival, he was bradycardic and hypotensive, but he was conscious, and both bradycardia and hypotension responded well to IV fluid and atropine. 12 leads electrocardiography (ECG) showed acute inferior STEMI complicated with 3 : 1 high-grade atrioventricular (AV) block (Figure 1(a)). He was transferred by EMS service to the hospital. CT scan of the head revealed no evidence of acute neurologic insult. Urgent coronary angiography showed a heavily thrombotic occlusion of proximal to mid right coronary artery (RCA). Thrombus aspiration was performed, then drug-eluting stent (DES) was deployed successfully. The final TIMI flow was TIMI II flow due to distal microembolization (Figures 2(a) and 2(b)). AV block directly resolved after coronary intervention. COVID-19 PCR testing was initially inconclusive then it came positive without COVID-19 infection related symptoms.

## 3. Case 2

A 55-year-old male has type 2 diabetes mellitus. He was admitted as case of symptomatic upper urinary tract infection; while he was in the emergency unit, he developed severe chest pain associated with hypotension. ECG showed sinus rhythm with acute inferior STEMI and first-degree AV block, subsequently; he developed complete heart block with signs of RV involvement in serial ECGs (Figure 1(b)).

He underwent thrombus aspiration and successful primary percutaneous coronary intervention (PPCI) to severe thrombotic stenosis from proximal to mid-RCA with DES (Figures 2(c) and 2(d)). Besides DAPT and anti-ischemic medications, he received 24 IV eptifibatide infusion.

Advanced AV block was resolved after IV fluids and inotropes while transferring him to catheterization laboratory. COVID-19 PCR test came positive without COVID-19 infection related symptoms.

## 4. Case 3

A 57-year-old man without positive cardiac or medical history presented with severe chest pain for 2 hours associated with dizziness and sweating. He has no history of fever or cough, but he has a history of general weakness for one week prior to the current event. On admission, vital signs were remarkable for a blood pressure of 89/56 mmHg, heart rate of 30 bpm, and oxygen saturation at 90% on room air, which improved to 97% with a nonrebreather mask. Initial ECG demonstrated sinus bradycardia with third-degree AV block and ST-segment elevation in inferior leads (Figure 1(c)). Coronary angiography revealed severe three-vessel diseases with significant distal left main stem stenosis. An attempt with balloon angioplasty was done to the thrombotic stenosis in mid-RCA in PPCI setting, but TIMI III flow was not achieved due to severe diffuse disease in distal RCA (Figure 2(e)). A transcutaneous temporary pacemaker wire was inserted. Chest radiography (CXR) demonstrated bilateral pulmonary congestion with infiltration in the left midlung zone. Transthoracic echocardiography showed severe global dysfunction with left ventricle ejection fraction (LVEF) 23%.

The cardiothoracic team was involved in the early stage for possible CABG once his medical condition is allowed, but he developed SARS-CoV-2 pneumonia associated with multiorgan failure in the background of active cardiac ischemia, he was intubated, and he was started on inotropes along with SARS-CoV-2 antiviral agents. COVID-19 pneumonia made his hospital course very challenging with many complications related to SARS-CoV-2 pneumonia like acute respiratory failure, disseminated intravascular coagulation DIC, severe sepsis, and septic shock with a superimposed bacterial infection. AV block was recovered over one week of admission, but due to the significant multiorgan failure, he was arrested and died after 3 weeks of admission.

## 5. Case 4

A 45-year-old gentleman presented with chest pain for more than 24 hours. Initial ECG showed ST-segment elevation with deep Q waves in inferior leads, which represented late presentation inferior. When he arrived in coronary care unit (CCU), he was hemodynamically stable without chest pain, subsequently, telemetry showed episodes of escape junctional bradycardia, isorhythmic AV dissociation (Figure 1(d)). Subsequently, ECG showed complete heart block (CHB). Coronary angiography showed 100% thrombotic stenosis in distal left coronary circumflex LCX (single vessel disease). Successful PCI to distal LCx with one drug-eluting stent (DES) (Figures 2(f) and 2(g)). COVID-19 PCR test came positive without COVID-19 infection related symptoms.

## 6. Case 5

A 50-year-old gentleman without significant medical history was brought by EMS with severe chest pain for 5 hours. Initial ECG showed ST-segment elevation in inferior leads with complete heart block which was resolved with 1 mg IV bolus atropine. Repeated ECG at emergency room still showed acute inferior STEMI with new right ventricular infarction.

Urgent coronary angiography demonstrated moderate stenosis in left anterior descending (LAD) and left circumflex (LCX) and totally thrombotic occlusion of midright coronary artery. Successful PPCI to mid-RCA with DES deployment without immediate complications (Figures 2(h) and 2(i)). COVID-19 PCR test came positive without COVID-19 infection related symptoms.

## 7. Case 6

A 36-year-old male without significant cardiac history is a smoker, one pack per day. He was brought by EMS due to central chest heaviness radiated to the left arm and associated with and profuse sweating. ECG showed escape junctional rhythm with complete heart block and acute ST-segment elevation in inferolateral leads. He was initially managed with atropine, IV fluid, noradrenaline IV infusion, and transcutaneous temporary pacing then transferred for PPCI.

**Figure 3:**
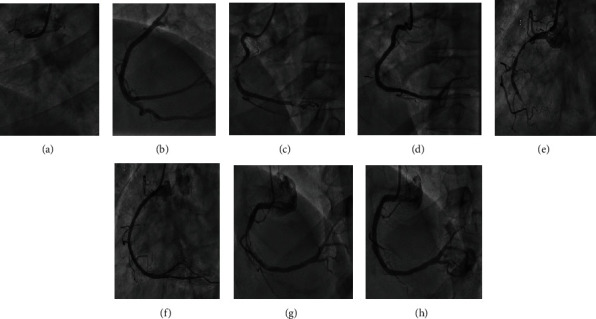
Coronary angiography to the culprit lesion. Case 6: (a) complete thrombotic occlusion in the very proximal segment of right coronary artery (RCA) with TIMI 0 flow; (b) successful PCI to proximal to mid-RCA with one drug-eluting stent DES, and TIMI III flow was achieved. Case 7: (c) severe thrombotic stenosis in mid-RCA with complete occlusion in the distal part due to distal embolization from the proximal thrombotic lesion; (d) successful thrombus aspiration and PCI to mid-RCA with one DES, and TIMI III flow was achieved distally. Case 8: (e) left anterior oblique (LAO) projection for right coronary artery (RCA) showed severe stenosis in the proximal segment followed by thrombotic occlusion from mid to distal RCA segment; (f) successful PCI to the proximal and distal RCA with 2 drug-eluting stent DES, and TIMI III flow was achieved. Case 9: (g) severe thrombotic stenosis in mid-RCA segment with TIMI III flow, a large thrombus burden in mid-RCA; (h) successful thrombus aspiration and PCI to RCA with 2 overlapping drug-eluting stent DES, and TIMI III flow was achieved.

Coronary angiography revealed 100% thrombotic stenosis in proximal RCA. Successful PPCI to ostial to proximal RCA with DES was done without immediate complications (Figures 3(a) and 3(b))). Bradycardia was improved with normal sinus rhythm after RCA coronary intervention. COVID-19 PCR test came positive without COVID-19 infection related symptoms.

## 8. Case 7

A 52-year-old male presented to the health center with sudden onset of chest pain for 2 hours. He was clinically stable, heart rate at 43 per minute, and blood pressure at 120/80 mmHg. Initial ECG showed sinus bradycardia, and ST-segment elevation in inferior leads associated with third-degree AV block (Figure 1(e)). He was given 300 mg aspirin, 300 mg clopidogrel, 30 mcg IV fentanyl, and 250 ml bolus of normal saline and then transferred to the heart center for PPCI. Coronary angiography revealed severe thrombotic stenosis in the mid-RCA; subsequently, thrombus aspiration was done, followed by successful DES deployment. The final TIMI flow was III (Figures 3(c) and 3(d)). COVID-19 PCR test came positive without COVID-19 infection related symptoms.

## 9. Case 8

A 49-year-old man with a history of hypertension presented with severe chest pain for 3 hours. He denies any history of fever or cough. Initial ECG demonstrated sinus rhythm with ST-segment elevation in inferior leads. On the way to the cath lab room, he developed hypotension and sinus bradycardia with complete heart block, which was responded to one dose of IV atropine 0.5 mg and IV fluid. Coronary angiography revealed severe three-vessel diseases with moderate distal left main stem stenosis. He underwent PPCI to thrombotic stenosis in proximal and distal RCA with 2 overlapping drug-eluting stents DES (Figures 3(e) and 3(f)). After coronary intervention, he maintained sinus rhythm without further requirements. Chest radiography (CXR) demonstrated no infiltration. Transthoracic echocardiography showed moderately global dysfunction with left ventricle ejection fraction (LVEF) 38%. COVID-19 PCR test came positive without COVID-19 infection related symptoms.

## 10. Case 9

A 57-year-old man with a history of uncontrolled type 2 diabetes mellitus was recently discharged from COVID-19 infection facility as a noncomplicated COVID-19 infection to continue the home quarantine. He presented with severe chest pain for 3 hours duration. ECG showed sinus rhythm with first-degree AV block PR interval of 340 milliseconds with ST-segment elevation in inferior leads (Figure 1(f)), but he was hemodynamically stable.

Coronary angiography demonstrated severe large burden thrombotic stenosis in mid-RCA, thrombus aspiration was done, followed by successful PPCI to the mid-RCA with 2 overlapping DES without immediate complications (Figures 3(g) and 3(h)).

First-degree AV block showed gradual regression and PR interval normalized during the stay in COVID-19 infection facility.

## 11. Discussion

Atrioventricular blocks are relatively common as a complication of acute myocardial ischemia; despite the marked improvements in the management of acute coronary syndromes, complete (AV) block can still be seen in the context of acute myocardial infarction (MI), especially inferior STEMIs.

The incidence rates of third-degree AV block in patients with inferior/posterior wall AMI were twice compared with patients with anterior wall AMI. Third-degree AV block in context of acute inferior wall STEMI is usually transient, supra-Hisian in origin, narrow QRS escape junctional rhythm, and usually the risk of mortality is low. Anterior wall infarctions, on the other hand, are usually infra-Hisian, have a wide QRS escape rhythm, and carry a much higher mortality rate because of the wide extension of myocardial damage [11].

In the prethrombolytic era, both Goldberg RJ et al. and Rathore SS et al. reported that the overall incidence of second- or third-degree AV block was recorded in 5% to 7% of patients presenting with acute MI [12, 13] and reached 28% in those with acute inferior STEMI [14]. In the thrombolytic era, no significant change in the rate of incidences of second- or third-degree AV block. It was recorded in 6.9% of patients presenting with acute inferior STEMI [15].

In the current modern era of primary percutaneous coronary intervention, the incidence and in-hospital mortality rate of complete atrioventricular block in patients with STEMI who underwent primary PCI was reduced compared to the data from the thrombolytic era, and the incidence of complete atrioventricular block (CAVB) dropped to 3.2 and 4.6% [16] and 3.2% [17] in 2 separate studies. The risk factors of CAVB are age, worse Killip class at presentation, female sex, smoking, hypertension, and diabetes [16.17].

COVID-19 outbreak has introduced new clinical and logistical challenges in the treatment of STEMI and related complications. We still have no clue about conduction abnormalities as one of the complications of STEMI in patients who tested positive for the COVID-19 infection regardless of COVID-19 infection symptoms.

All patients who presented with inferior MI without evidence of viral infection did not develop any type of atrioventricular block, while all documented cases with complications related to conduction abnormalities in the settings of inferior MI have tested positive for the COVID-19 infection.

The factors that can stand behind the high incidence of AV block (61%) are theoretically due to:

1. Some STEMI patients were presented to the hospital later than usual; this could be due to the new policies that took place over the country to minimize the risk of COVID infection transmission and spread like the lockdown areas
2. Patient fears of getting the COVID-19 infection if they seek medical help, assuming that the risk of infection is higher in places that provide health care like hospitals. It is known that stress and anxiety can trigger cardiovascular problems like heart attacks. It is no doubt that the COVID-19 outbreak carries lots of social and financial negative impacts that could lead to an increase in the risk of cardiovascular events and related complications in general
3. The COVID-19 infection itself might be associated with larger MI extension, high burden clots, and more active inflammatory process, which can be measured by elevated CRP, ferritin, and IL6
4. High burden clots also could be a potential factor. Four patients (22%) underwent thrombus aspiration despite the current international guidelines do not support the routine thrombus aspiration in the context of acute STEMI. It is worldwide accepted that thromboembolic events and pulmonary embolism are common in very critical COVID-19 patients. Most of the current regimens encourage the use of anticoagulation as part of management protocols with antiviral agents, especially in the high-risk patients [18]

We also noted that patient's age was lower than what was watched previously in Meine et al. study [15] even all of them were male with lower Killip class at presentation. In most cases, AV block recovered with conservative or invasive treatments, and only one patient required a transvenous temporary pacemaker.

Only one patient (less than 1% of the total) died because of multiorgan failure caused by COVID-19 infection complications in the context of acute inferior STEMI.

## 12. Conclusion

Based on our findings, the incidence of atrioventricular blocks is higher than the incidence of AV block even in the prethrombolytic era. In almost 24 months of COVID-19 outbreaks worldwide, we are still lacking understanding of the aspects of COVID-19 behavior. While more recent studies provide more details of how the infection invades body organs in general, the cardiac manifestations of COVID-19 infections are still unclear, especially those related to the conduction system. The higher rate of patients with inferior STEMI who tested positive for COVID-19 infection developed conduction system abnormalities, even double the incidence of such a complication in the prethrombolytic era. Further studies with larger numbers are recommended to clarify why the incidence of AV blocks is higher in COVID-19 infection patients.

## Figures and Tables

**Table 1:** The case series summary including the presentation symptoms, coronary angiography, and ECG findings.

Patient	Presentation	PPCI is done in PPCI setting	Culprit coronary artery	Symptoms related to COVID-19 infection	The peak troponin *T* (ng/L)	Type of AV block	Specific treatment to AV block	Outcomes
1	Acute inferior STEMI	No	Right coronary artery (RCA)	None	11979	Mobitz 2 type 2 AV block	Atropine and urgent PCI	Recovered
2	Acute inferior STEMI with RV infarction	Yes	RCA	None	9740	Complete heart block	Dopamine and fluid	Recovered
3	Acute inferior STEMI	No	RCA	Severe COVID-19 pneumonia	9704	Complete heart block	Transvenous temporary pacing	Recovered patient died
4	Late inferior STEMI	No	Left coronary circumflex artery (LCX)	None	7226	Accelerated junctional bradycardia, isorhythmic AV dissociation, and complete heart block	Urgent PCI	Recovered
5	Acute inferior STEMI with RV infarction	Yes	RCA	None	3763	Complete heart block	Atropine	Recovered
6	Acute inferolateral STEMI	Yes	RCA	None	11926	Complete heart block	Atropine, transcutaneous temporary pacing, and PPCI	Recovered
7	Acute inferior STEMI	Yes	RCA	None	2320	Complete heart block	PPCI	Recovered
8	Acute inferior STEMI	Yes	RCA	None	12442	Complete heart block	Atropine	Recovered
9	Acute inferior STEMI	Yes	RCA	STEMI happened while he was in the quarantine	1112	First-degree AV block	PPCI	Recovered
